# Supplementary material for: Time-series transcriptome analysis identified differentially expressed genes in broiler chicken infected with mixed Eimeria species
Source: Front Genet. 2022 Aug 8;13:886781. doi: 10.3389/fgene.2022.886781 (PMC9393255; doi:10.3389/fgene.2022.886781)
Supplement: Supplementary file 2 [file DataSheet1.ZIP › 7dpi_GO.Gsea.1625106495923/GOBP_MITOCHONDRIAL_RESPIRATORY_CHAIN_COMPLEX_ASSEMBLY.html]

Details for gene set GOBP\_MITOCHONDRIAL\_RESPIRATORY\_CHAIN\_COMPLEX\_ASSEMBLY[GSEA]

|  || Dataset | TMM\_7dpi\_gct\_format\_7dpi\_gct\_format.Class\_4dpi.cls #PC\_versus\_NC.Class\_4dpi.cls #PC\_versus\_NC\_repos |
| Phenotype | Class\_4dpi.cls#PC\_versus\_NC\_repos |
| Upregulated in class | 1 |
| GeneSet | GOBP\_MITOCHONDRIAL\_RESPIRATORY\_CHAIN\_COMPLEX\_ASSEMBLY |
| Enrichment Score (ES) | 0.6597478 |
| Normalized Enrichment Score (NES) | 3.1799705 |
| Nominal p-value | 0.0 |
| FDR q-value | 0.0 |
| FWER p-Value | 0.0 |
Table: GSEA Results Summary

  

Fig 1: Enrichment plot: GOBP\_MITOCHONDRIAL\_RESPIRATORY\_CHAIN\_COMPLEX\_ASSEMBLY      
 Profile of the Running ES Score & Positions of GeneSet Members on the Rank Ordered List

  

| SYMBOL | TITLE | RANK IN GENE LIST | RANK METRIC SCORE | RUNNING ES | CORE ENRICHMENT || 1 | COA3 | na | 42 | 1.483 | 0.0376 | Yes |
| 2 | COX14 | na | 85 | 1.303 | 0.0702 | Yes |
| 3 | NDUFAF3 | na | 148 | 1.148 | 0.0968 | Yes |
| 4 | SCO2 | na | 194 | 1.052 | 0.1221 | Yes |
| 5 | NDUFA2 | na | 303 | 0.935 | 0.1390 | Yes |
| 6 | NDUFB6 | na | 307 | 0.933 | 0.1646 | Yes |
| 7 | NDUFS5 | na | 308 | 0.932 | 0.1904 | Yes |
| 8 | NDUFB7 | na | 323 | 0.915 | 0.2146 | Yes |
| 9 | NDUFB2 | na | 348 | 0.885 | 0.2371 | Yes |
| 10 | NDUFAB1 | na | 356 | 0.876 | 0.2608 | Yes |
| 11 | NDUFA7 | na | 371 | 0.857 | 0.2834 | Yes |
| 12 | NDUFC2 | na | 394 | 0.834 | 0.3046 | Yes |
| 13 | SDHAF3 | na | 490 | 0.751 | 0.3175 | Yes |
| 14 | NDUFA11 | na | 497 | 0.746 | 0.3377 | Yes |
| 15 | SDHAF4 | na | 516 | 0.730 | 0.3564 | Yes |
| 16 | NDUFA13 | na | 557 | 0.708 | 0.3726 | Yes |
| 17 | NDUFS3 | na | 623 | 0.675 | 0.3859 | Yes |
| 18 | NDUFB3 | na | 656 | 0.657 | 0.4014 | Yes |
| 19 | NDUFS8 | na | 661 | 0.653 | 0.4192 | Yes |
| 20 | NDUFS6 | na | 686 | 0.642 | 0.4349 | Yes |
| 21 | UQCC3 | na | 735 | 0.620 | 0.4481 | Yes |
| 22 | NDUFA1 | na | 742 | 0.616 | 0.4646 | Yes |
| 23 | NDUFA5 | na | 794 | 0.593 | 0.4768 | Yes |
| 24 | NDUFAF8 | na | 894 | 0.557 | 0.4839 | Yes |
| 25 | NDUFB5 | na | 902 | 0.554 | 0.4987 | Yes |
| 26 | SDHAF2 | na | 919 | 0.550 | 0.5126 | Yes |
| 27 | NDUFV3 | na | 988 | 0.527 | 0.5215 | Yes |
| 28 | NDUFB4 | na | 1015 | 0.517 | 0.5336 | Yes |
| 29 | NDUFA12 | na | 1023 | 0.513 | 0.5473 | Yes |
| 30 | NDUFA6 | na | 1079 | 0.492 | 0.5563 | Yes |
| 31 | NDUFA8 | na | 1080 | 0.492 | 0.5699 | Yes |
| 32 | NDUFS2 | na | 1124 | 0.477 | 0.5795 | Yes |
| 33 | NUBPL | na | 1139 | 0.472 | 0.5914 | Yes |
| 34 | NDUFB10 | na | 1215 | 0.450 | 0.5976 | Yes |
| 35 | UQCRFS1 | na | 1263 | 0.437 | 0.6058 | Yes |
| 36 | NDUFV1 | na | 1307 | 0.428 | 0.6141 | Yes |
| 37 | NDUFB8 | na | 1330 | 0.423 | 0.6239 | Yes |
| 38 | NDUFS7 | na | 1371 | 0.412 | 0.6320 | Yes |
| 39 | NDUFV2 | na | 1395 | 0.408 | 0.6414 | Yes |
| 40 | ACAD9 | na | 1483 | 0.385 | 0.6447 | Yes |
| 41 | ECSIT | na | 1497 | 0.382 | 0.6542 | Yes |
| 42 | SMIM20 | na | 1565 | 0.363 | 0.6587 | Yes |
| 43 | NDUFB9 | na | 1842 | 0.299 | 0.6438 | Yes |
| 44 | NDUFB1 | na | 1995 | 0.272 | 0.6386 | Yes |
| 45 | CHCHD4 | na | 2045 | 0.263 | 0.6418 | Yes |
| 46 | SAMM50 | na | 2097 | 0.254 | 0.6446 | Yes |
| 47 | SCO1 | na | 2199 | 0.237 | 0.6427 | Yes |
| 48 | BCS1L | na | 2225 | 0.232 | 0.6470 | Yes |
| 49 | TIMM21 | na | 2269 | 0.226 | 0.6497 | Yes |
| 50 | TFAM | na | 2295 | 0.223 | 0.6537 | Yes |
| 51 | TAZ | na | 2298 | 0.223 | 0.6597 | Yes |
| 52 | NDUFAF6 | na | 2404 | 0.207 | 0.6567 | No |
| 53 | COX19 | na | 2461 | 0.197 | 0.6574 | No |
| 54 | UQCC1 | na | 2548 | 0.185 | 0.6554 | No |
| 55 | COA5 | na | 2793 | 0.151 | 0.6391 | No |
| 56 | NDUFA9 | na | 2879 | 0.141 | 0.6359 | No |
| 57 | NDUFA10 | na | 3412 | 0.073 | 0.5933 | No |
| 58 | COX17 | na | 3545 | 0.059 | 0.5839 | No |
| 59 | COX16 | na | 3738 | 0.036 | 0.5688 | No |
| 60 | NDUFS1 | na | 3763 | 0.033 | 0.5677 | No |
| 61 | TMEM126A | na | 3795 | 0.029 | 0.5659 | No |
| 62 | TIMMDC1 | na | 3850 | 0.022 | 0.5620 | No |
| 63 | NDUFAF1 | na | 3921 | 0.014 | 0.5565 | No |
| 64 | SURF1 | na | 3926 | 0.013 | 0.5565 | No |
| 65 | NDUFS4 | na | 4402 | -0.040 | 0.5178 | No |
| 66 | COX18 | na | 4742 | -0.075 | 0.4915 | No |
| 67 | TTC19 | na | 4743 | -0.075 | 0.4936 | No |
| 68 | SLC25A33 | na | 5797 | -0.179 | 0.4103 | No |
| 69 | NDUFAF5 | na | 5835 | -0.184 | 0.4123 | No |
| 70 | OMA1 | na | 6042 | -0.206 | 0.4007 | No |
| 71 | FOXRED1 | na | 6248 | -0.229 | 0.3899 | No |
| 72 | COX20 | na | 6348 | -0.239 | 0.3882 | No |
| 73 | IMMP2L | na | 6710 | -0.275 | 0.3656 | No |
| 74 | AIFM1 | na | 7048 | -0.314 | 0.3460 | No |
| 75 | NDUFAF4 | na | 7678 | -0.381 | 0.3039 | No |
| 76 | LYRM7 | na | 8350 | -0.465 | 0.2605 | No |
| 77 | NDUFAF2 | na | 8545 | -0.491 | 0.2579 | No |
| 78 | NDUFAF7 | na | 8945 | -0.541 | 0.2394 | No |
| 79 | FASTKD3 | na | 9468 | -0.626 | 0.2130 | No |
Table: GSEA details [plain text format]

  

Fig 2: GOBP\_MITOCHONDRIAL\_RESPIRATORY\_CHAIN\_COMPLEX\_ASSEMBLY      
 Blue-Pink O' Gram in the Space of the Analyzed GeneSet

  

Fig 3: GOBP\_MITOCHONDRIAL\_RESPIRATORY\_CHAIN\_COMPLEX\_ASSEMBLY: Random ES distribution      
 Gene set null distribution of ES for **GOBP\_MITOCHONDRIAL\_RESPIRATORY\_CHAIN\_COMPLEX\_ASSEMBLY**
